# Construct of qualitative diagnostic biomarkers specific for glioma by pairing serum microRNAs

**DOI:** 10.1186/s12864-023-09203-w

**Published:** 2023-03-02

**Authors:** Hongdong Li, Liyuan Ma, Fengyuan Luo, Wenkai Liu, Na Li, Tao Hu, Haijian Zhong, You Guo, Guini Hong

**Affiliations:** 1 School of Medical Information Engineering, Gannan Medical University, Ganzhou, 341000 China; 2 Medical Big Data and Bioinformatics Research Centre at First Affiliated Hospital of Gannan Medical University, Ganzhou, 341000 China

**Keywords:** Serum microRNA, Glioma, Diagnostic biomarker, Relative expression ordering

## Abstract

**Background:**

Serum microRNAs (miRNAs) are promising non-invasive biomarkers for diagnosing glioma. However, most reported predictive models are constructed without a large enough sample size, and quantitative expression levels of their constituent serum miRNAs are susceptible to batch effects, decreasing their clinical applicability.

**Methods:**

We propose a general method for detecting qualitative serum predictive biomarkers using a large cohort of miRNA-profiled serum samples (*n* = 15,460) based on the within-sample relative expression orderings of miRNAs.

**Results:**

Two panels of miRNA pairs (miRPairs) were developed. The first was composed of five serum miRPairs (5-miRPairs), reaching 100% diagnostic accuracy in three validation sets for distinguishing glioma and non-cancer controls (*n* = 436: glioma = 236, non-cancers = 200). An additional validation set without glioma samples (non-cancers = 2611) showed a predictive accuracy of 95.9%. The second panel included 32 serum miRPairs (32-miRPairs), reaching 100% diagnostic performance in training set on specifically discriminating glioma from other cancer types (sensitivity = 100%, specificity = 100%, accuracy = 100%), which was reproducible in five validation datasets (*n* = 3387: glioma = 236, non-glioma cancers = 3151, sensitivity> 97.9%, specificity> 99.5%, accuracy> 95.7%). In other brain diseases, the 5-miRPairs classified all non-neoplastic samples as non-cancer, including stroke (*n* = 165), Alzheimer’s disease (*n* = 973), and healthy samples (*n* = 1820), and all neoplastic samples as cancer, including meningioma (n = 16), and primary central nervous system lymphoma samples (*n* = 39). The 32-miRPairs predicted 82.2 and 92.3% of the two kinds of neoplastic samples as positive, respectively. Based on the Human miRNA tissue atlas database, the glioma-specific 32-miRPairs were significantly enriched in the spinal cord (*p* = 0.013) and brain (*p* = 0.015).

**Conclusions:**

The identified 5-miRPairs and 32-miRPairs provide potential population screening and cancer-specific biomarkers for glioma clinical practice.

**Supplementary Information:**

The online version contains supplementary material available at 10.1186/s12864-023-09203-w.

## Background

Glioma is adults’ most common primary brain tumor, accounting for approximately 80% of all central nervous system malignant tumors [1]. Due to its specific development site and late diagnosis, the 5-year survival rate is about 3% [2]. Identifying effective early diagnostic biomarkers is crucial to improving the prognosis of glioma. Currently, the diagnosis of glioma that guides clinical practice is mainly accomplished by biopsy or tissue obtained after tumor resection [3]. However, this surgical approach is invasive, and it is difficult to get sufficient tumor material in deep or surgically inaccessible tumors. Magnetic resonance imaging is the preferred non-invasive method for diagnosing glioma. However, in addition to being expensive and cumbersome, its diagnostic information is preliminary [4]. Therefore, developing non-invasive methods for diagnosing glioma tumors remains a challenge, especially regarding specific biomarkers.

MicroRNAs (miRNAs) have been reported to play essential roles in different pathological and physiological processes, including cancer development [5]. Many studies have used serum miRNAs as predictors for the diagnosis and prognosis of glioma, demonstrating the potential of serum miRNAs in serving as non-invasive biomarkers [6–8]. For example, the expression of serum *miR-100* [6] and *miR-29b* [8] has been used to discriminate glioblastoma patients from healthy controls. A recent study explored whether serum miRNAs could detect glioma and differentiate between glioblastoma, primary central nervous system lymphoma (PCNSL), and metastatic brain tumors [9]. Their diagnostic models were also constructed based on composite scores of miRNA expression levels. Such quantitative expression levels-based approaches suffer from several shortcomings. First, the expression levels of miRNAs may vary significantly due to technical fluctuations and batch effects [10]. Second, the expression of specific miRNAs may differ between other races and regions due to the heterogeneity of individual genetics. Once the miRNA expressions fluctuate, the patients’ classification may be biased. Therefore, such biomarkers may often fail in independent samples from different cohorts. Moreover, like the standardization of data, preprocessing is also required when applying quantitative expression levels-based biomarkers, making them difficult to apply to individual clinical practice [11]. Another problem is current serum miRNA biomarkers for glioma still cannot have satisfactory diagnostic accuracy. For example, the diagnostic area under the curve for serum *miR-100* was 0.839, with a sensitivity and specificity of 83.33 and 77.89%, respectively [6]. The area under the curve for *miR-29b* was 0.866 (sensitivity = 83.18%, specificity = 81.25%) [8]. Also, previous studies lacked independent large sample size validations, which may hinder their applicability.

To overcome the limitations mentioned above and identify robust miRNA biomarkers for screening and specific glioma detection, we conducted an extensive case study containing 15,460 serum samples involving 13 cancer types and non-cancer control samples, and four other brain diseases and healthy samples. The relative expression orderings (REOs) of serum miRNAs were employed in developing predictive models, which belonged to the kind of single sample classifier [12]. Compared to the quantitative expression levels, REO-based biomarkers are insensitive to batch effects, data normalization methods, partial RNA degradation, and RNA amplification bias [13]. Therefore, we developed two models based on within-sample REOs of serum miRNAs using large samples (*n* = 15,460). The first model was used to differentiate between glioma and non-cancer control samples. The second model was used to discriminate gliomas from other cancers specifically. Prior to this, few studies have identified glioma-specific diagnostic biomarkers from serum. Considering the clinical ease of use of serum material and the stability and robustness of REO-based biomarkers, the two models we developed would have the potential to provide additional benefits for glioma screening and specific diagnosis in clinical practice.

## Methods

### Data source and data preprocessing

We downloaded six cancer datasets of serum miRNA expression from the GEO database (http://www.ncbi.nlm.nih.gov/geo/), with a total of 12,447 samples, of which 8032 were non-cancer control samples, and 4415 were serum samples of 13 cancer types (Detailed information in Table 1). We also collected three datasets of serum miRNA expression for four brain diseases: stroke (GSE117064; *n* = 1785: stroke = 173, healthy controls = 1612), Alzheimer’s disease (GSE120584; *n* = 1309: Alzheimer’s disease = 1021, healthy controls = 288), meningioma and PCNSL (GSE139031; *n* = 59: meningioma = 17, PCNSL = 42). These data were assayed by the 3D-Gene Human miRNA V21_1.0.0 platform, detecting a total of 2550 miRNAs. GSE113486 was used as the training set, and all other datasets were used as the validation set.

**Table 1:** Data analyzed in this study

Phenotype	GSE113486 [14]	GSE112264 [15]	GSE113740 [16]	GSE106817 [17]	GSE139031 [9]	GSE122497 [18]
non-cancer	100(97)^a^	41(39)	10(10)	2759(2611)	157(151)	4965(4720)
Biliary Tract Cancer	40(39)	50(48)	25(25)	–	–	–
Bladder Cancer	392(370)	50(48)	25(24)	–	–	–
Breast Cancer	40(37)	–	25(24)	115(111)		
Colorectal Cancer	40(38)	50(48)	25(24)	115(107)	–	–
Esophageal Cancer	40(39)	50(48)	25(24)	88(83)		566(542)
Gastric Cancer	40(39)	50(49)	25(24)	115(110)	–	–
Glioma	40(38)	50(48)	25(24)	–	170(164)	–
Hepatocellular Carcinoma	40(39)	50(47)	40(39)	81(76)	–	–
Lung Cancer	40(37)	50(49)	25(23)	115(109)	–	–
Ovarian Cancer	40(37)	–	25(23)	320(306)	–	–
Pancreatic Cancer	40(39)	50(46)	25(24)	115(110)	–	–
Prostate Cancer	40(38)	809(773)	25(24)	–	–	–
Sarcoma	40(38)	50(48)	4(4)	115(111)	–	–

^a^The number in the bracket indicates the remaining sample size after removing outlier samples

To ensure the reliability of the data, we removed outlier samples from each phenotype in each dataset by the following criteria. First, the correlation coefficient between the expression levels of miRNAs of any two samples was calculated. If the mean value of the correlation coefficients between one sample and other samples was outside twice the standard deviation of the mean value of all samples, the sample was considered an outlier sample and removed from the dataset.

### Discovery of relative expression ordering-based biomarkers

**Fig. 1:**
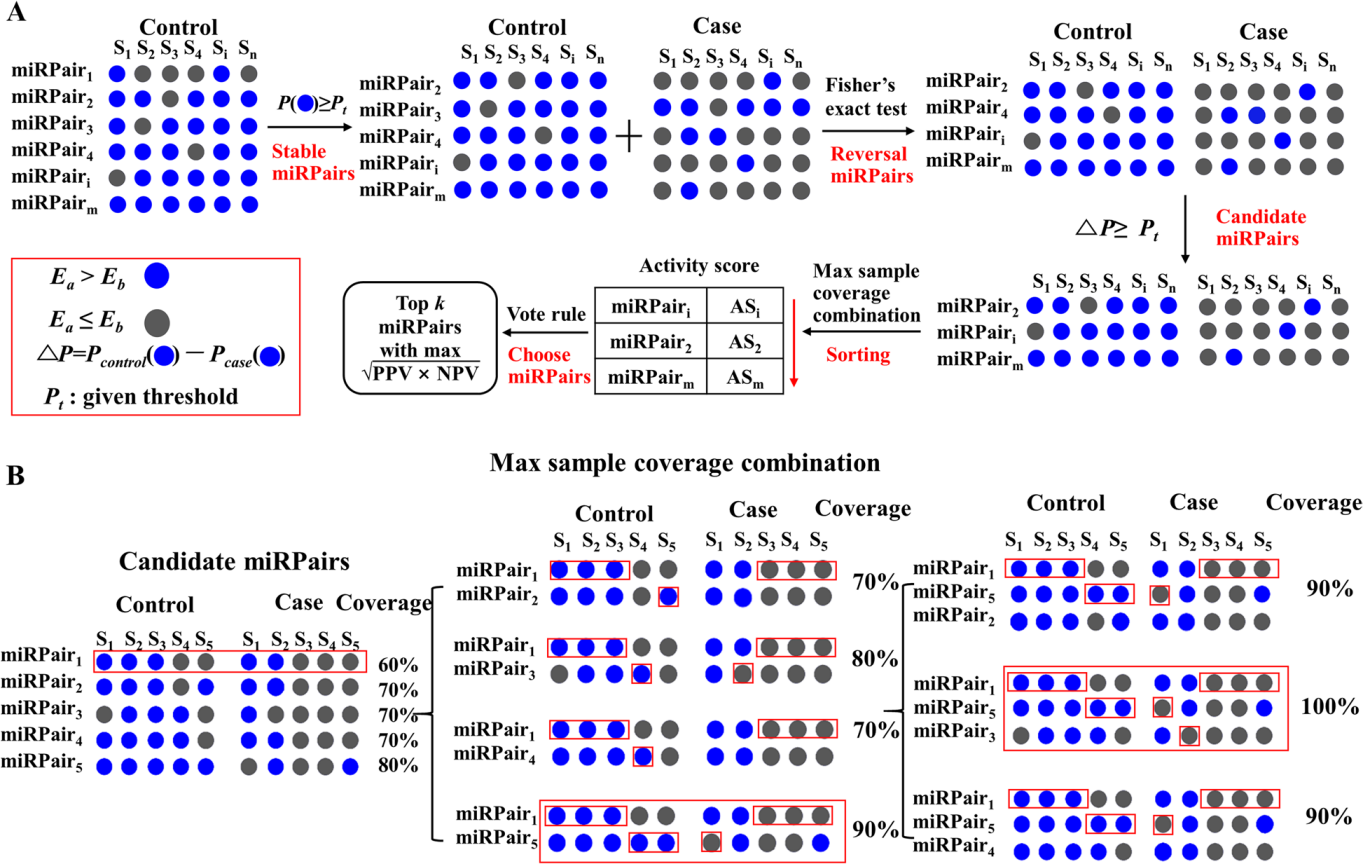
**A** Flow chart of the detection of the biomarkers; **B** The identification of a combination with maximum sample coverage

Given a training set containing control and case samples, we defined the REO of two miRNAs within a sample as *E*_*miRNAa*_ > *E*_*miRNAb*_ or *E*_*miRNAa*_ ≤ *E*_*miRNAb*_. Suppose a significant difference exists in the distribution of REOs of a miRNA pair (miRPair) between the control and case samples. The REO of the miRPair can be naturally used to predict to which group an unknown sample belongs. Based on this hypothesis, the flow of identifying REO-based biomarkers is as follows (Fig. 1A).

### Detection of stable and reversed miRNA pairs

The miRNAs detected in the training set were paired to form *n*(*n-1*) miRPairs. Then, for a miRPair (*miRNA*_*a*_, *miRNA*_*b*_), the percentage *P* of its REO exhibiting as *E*_*miRNAa*_ *> E*_*miRNAb*_ in the control samples was calculated as follows,

$$P\left({E}_{miRNAa}>{E}_{miRNAb}\right)=k/m\times 100\%,$$

where *k* represents the number of those samples with *E*_*miRNAa*_ *> E*_*miRNAb*_ in control samples, and *m* represents the total number of control samples.

Suppose the percentage *P* of a miRPair in the control samples is no less than a threshold, for example, 95%, which is adjustable as needed. Then, the REO of the miRPair is considered stable in the control samples and is called a stable miRPair.

For each stable miRPair, the numbers of control and case samples showing the REOs of *E*_*miRNAa*_ *> E*_*miRNAb*_ and *E*_*miRNAa*_ ≤ *E*_*miRNAb*_ were calculated and denoted by *n*_*1*_ and *n*_*2*_, *m*_*1*_ and *m*_*2*_, respectively. Fisher’s exact test was used to test whether the distribution of REOs of the miRPair in the controls differed from that in the case samples. If the BH adjusted *p*-value is smaller than 5%, the REO of the miRPair was considered significantly reversed under the case condition and defined as a reversed miRPair.

### Determination of candidate miRPairs

For a reversed miRPair, △*P* was calculated, where △*P*=*P*_*control*_(*E*_*miRNAa*_ *> E*_*miRNAb*_)*-P*_*case*_(*E*_*miRNAa*_ *> E*_*miRNAb*_). The greater the △*P* value, the more significant the difference in the distributions of REOs between control and case samples. For a miRPair, △*P* = *1* indicates that the REOs of the miRPair in control samples are all *E*_*miRNAa*_ *> E*_*miRNAb*_, while in case samples are all *E*_*miRNAa*_ ≤ *E*_*miRNAb*_. Those miRPairs satisfying △*P* > *P*_*t*_ (*P*_*t*_ is a given threshold) were determined as candidate miRPairs.

### Calculation of activity scores of candidate miRPairs

We identified a combination with maximum sample coverage for each candidate miRPair. The steps were as follows.

First, we defined the reference pattern of REO as showing *E*_*miRNAa*_ *> E*_*miRNAb*_ under the control condition or *E*_*miRNAa*_ ≤ *E*_*miRNAb*_ under the case condition. The combination covered a sample if at least one miRPair it possessed exhibited the reference pattern on that sample. Sample coverage was then calculated as the percentage of covered samples to the total sample number in the training set.

Then, we searched for a combination with the maximum sample coverage for each candidate miRPair. For a candidate *miRPair*_*i*_, at first, the combination is {*miRPair*_*i*_}. The search process is shown in Fig. 1B and is described as follows. Except for miRPair(s) already in the combination, each remaining candidate was separately added to the combination, and the corresponding sample coverage was calculated. The combination {*miRPair*_*i*_, *miRPair*_*j*_} with the most extensive sample coverage was selected. Then, the search process for the next miRPair is kept on from the remaining candidates until adding a miRPair into the combination cannot increase the sample coverage. The obtained combination is the maximum sample coverage combination identified for the candidate *miRPair*_*i*_.

After searching combinations with the maximum sample coverage, we can then define an activity score for each candidate miRPair, i.e., the number of occurrences in all combinations. The higher the active score, the greater the importance of the candidate miRPair.

### Determination of final REO-based biomarkers

The candidate miRPairs are sorted according to their activity scores, from the largest to the smallest. The top *k* miRPairs are taken as prediction biomarkers, respectively, where *k* = 1: *n* (*n* is the number of candidate miRPairs). Then, the prediction models are constructed based on the voting rules according to the REOs of miRPairs in the model. The geometric mean of negative predictive value (NPV) and positive predictive value (PPV) is calculated, and the top *k* miRPairs that reach the maximum first are used as the final prediction biomarker.

### Differential miRNA identification and functional analysis

Differentially expressed miRNAs between phenotypes were identified using the Student’s *t*-test and were considered significant at a false discovery rate (FDR) smaller than 5%.

The miRNA functional annotation was based on miEAA online miRNA functional enrichment and annotation tool [19], which can automatically predict the target mRNAs for identified miRNAs and perform functional enrichment analysis. The miRNAs involved in miRPairs were analyzed using the Tissue Atlas database for tissue-specific expression enrichment analysis [20] and the KEGG database [21] for pathway enrichment analysis.

### Statistical analysis

All statistical analyses in this study were performed with R 3.6.1 software.

## Results

### Serum diagnostic model of 5-miRPairs for glioma

The 97 non-cancer control and 38 glioma serum samples in GSE113486 were used as control and case samples for training. With *P*(*E*_*miRNAa*_ *> E*_*miRNAb*_) ≥ 95% in non-cancer control samples, we identified a total of 1,337,295 stable miRPairs, and 94.63% (1,265,546) of them still maintained the REOs of *E*_*miRNAa*_ *> E*_*miRNAb*_ in 95% of the 4720 non-cancer samples in GSE122497. This result indicated that the within-sample REOs of miRNAs had high stability.

Then, we assessed the REO alterations in glioma serum. Among the 1,265,546 stable miRPairs, 857,298 showed a significant reversal of REOs in the serum of glioma (FDR < 5%, Fisher’s exact test). We found that 34 reversed miRPairs showed an REO completely reversed between non-cancer and glioma, i.e., all showed *E*_*miRNAa*_ *> E*_*miRNAb*_ in non-cancer samples, while in glioma, all showed *E*_*miRNAa*_ *≤ E*_*miRNAb*_ (Δ*P* = 1, see Methods), indicating that all 34 of them can be predictive biomarkers. For simplicity, in the natural order, the first five of the 34 significantly reversed miRPairs were selected to construct the glioma prediction model. The five miRPairs were (*hsa-miR-125a-3p*, *hsa-miR-1914-5p*), (*hsa-miR-125a-3p*, *hsa-miR-3162-3p*), (*hsa-miR-887-3p*, *hsa-miR-1225-3p*), (*hsa-miR-1203*, *hsa-miR-1470*), and (*hsa-miR-1203*, *hsa-miR-7108-3p*), referred to as 5-miRPairs.

A model of prediction by majority voting based on the 5-miRPairs was developed. A sample was predicted as non-cancer control if more than three miRPairs exhibited a pattern of *E*_*miRNAa*_ *> E*_*miRNAb*_, and vice versa for glioma samples. In the three independent validation sets of GSE13901, GSE112264, and GSE113740, the prediction accuracy was 100% (Table 2). The GSE106817 included no glioma samples, and only the 2611 non-cancer samples were predicted, with an accuracy of 95.90%. This result indicates the excellent prediction efficacy of the 5-miRPairs model for classifying glioma and non-cancer samples.

**Table 2:** The performance of the predictive models in independent validation datasets

**Dataset**	**#Non-cancer**	**#Glioma**	**SEN%**	**SPE%**	**ACC%**	**PPV%**	**NPV%**
5-miRPairs							
GSE139031	151	164	100%	100%	100%	100%	100%
GSE112264	39	48	100%	100%	100%	100%	100%
GSE113740	10	24	100%	100%	100%	100%	100%
GSE106817	2611	–	–	–	95.90%	–	–
**Dataset**	**#Non-glioma**	**#Glioma**	**SEN%**	**SPE%**	**ACC%**	**PPV%**	**NPV%**
32-miRPairs							
GSE112264	1204	48	97.92%	99.58%	98.74%	90.38%	99.91%
GSE113740	282	24	100%	100%	100%	100%	100%
GSE106817	1123	–	–	–	98.31%	–	–
GSE139031	–	164	–	–	95.73%	–	–
GSE122497	542	–	–	–	99.45%	–	–

*Abbreviation*: *SEN* sensitivity, *SPE* specificity, *ACC* accuracy, *PPV* positive predictive value, *NPV* negative predictive value

### Low cancer specificity of 5-miRPairs for glioma

We predicted other cancer types based on the same majority voting rule to verify whether the 5-miRPairs model was glioma-specific. The model averagely grouped 95.70% of the 12 cancer types in GSE113486 to glioma (Table 3). In GSE112264, an average of 95.20% of the ten cancer types were predicted as glioma. Similarly, 95.80% of the 12 cancer types in GSE13470 were classified as glioma, suggesting that the model is not glioma specific.

**Table 3:** The performance of 5-miRPairs in predicting other cancer types

Cancer type	GSE113486		GSE112264		GSE113740	
	No.	Percentage^a^	No.	Percentage	No.	Percentage
Bladder Cancer	370	91.08%	48	87.50%	24	95.83%
Breast Cancer	37	94.59%	–	–	24	95.83%
Biliary Tract Cancer	39	94.87%	48	95.83%	25	96.00%
Colorectal Cancer	38	89.47%	48	93.75%	24	87.50%
Esophageal Cancer	39	100%	48	100%	24	100%
Gastric Cancer	39	100%	49	95.92%	24	100%
Hepatocellular Carcinoma	39	100%	47	100%	39	100%
Lung Cancer	37	100%	49	97.96%	23	100%
Ovarian Cancer	37	94.59%	–	–	23	95.65%
Pancreatic Cancer	39	100%	46	100%	24	100%
Prostate Cancer	38	84.21%	773	85.25%	24	83.33%
Sarcoma	38	100%	48	95.83%	–	–

^a^The percentage of samples predicted to be positive

**Fig. 2:**
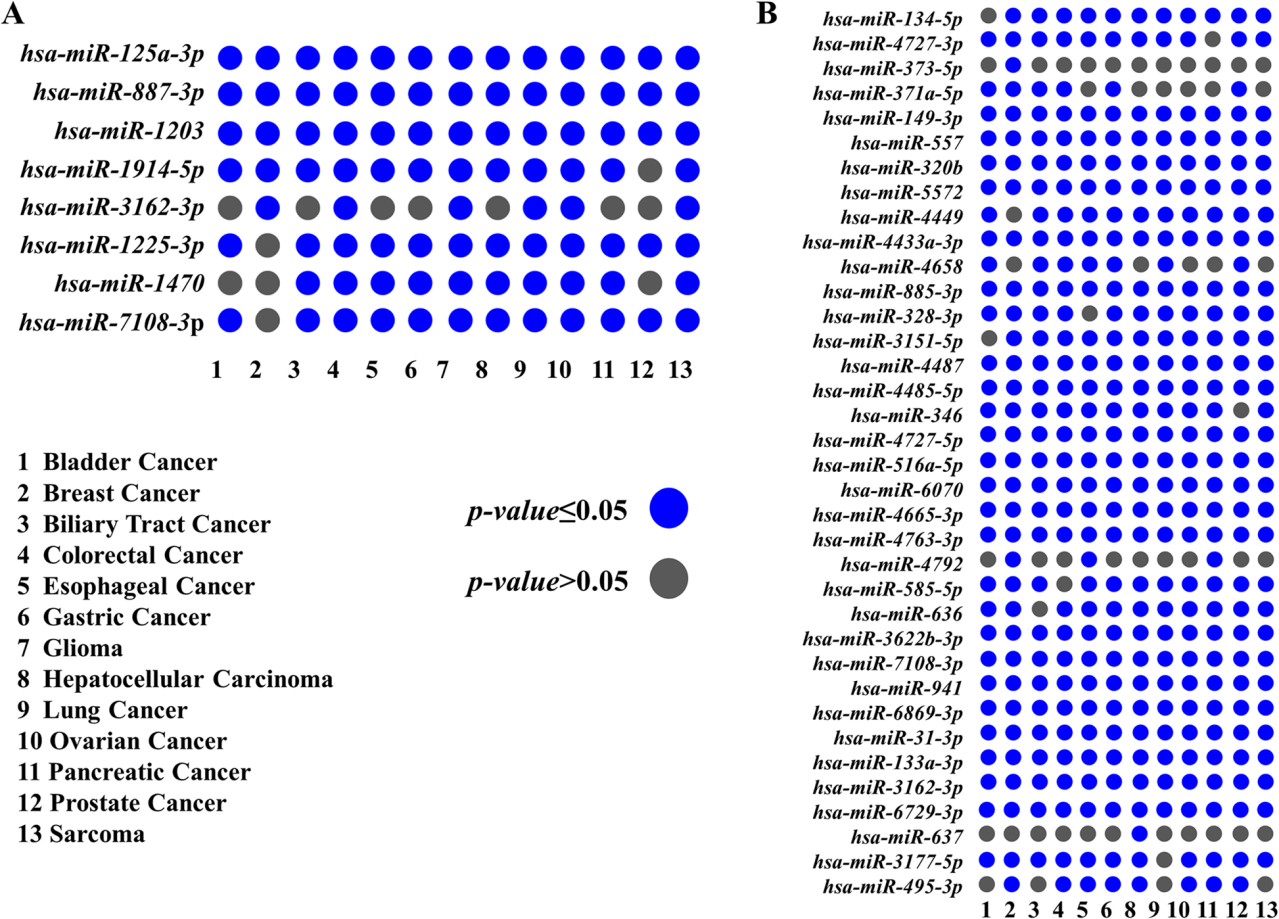
Differential analysis of miRNAs in the models. **A** Differential expression of 5-miRPairs in 13 cancer types compared to non-cancer controls; **B** Differential expression of 32-miRPairs in 12 cancer types compared to glioma samples

We analyzed the reasons for the low glioma specificity of the 5-miRPairs model from the perspective of differential miRNAs. In the training set, a comparison of the miRNA expression in the 13 cancers with non-cancer control samples revealed that each of the eight miRNAs involved in the model was differentially expressed in at least six cancers (Fig. 2A). The average number of differential miRNAs per cancer was 7 ± 1.08, with *hsa-miR-125a-3p*, *hsa-miR-887-3p*, and *hsa-miR-1203* being differentially expressed in all 13 cancer types. Studies reported that these three miRNAs are associated with cancerogenesis and progression in multiple cancers [22–24]. This result suggests common alterations in serum miRNAs in different cancer types.

### Glioma-specific serum diagnostic model of 32-miRPairs

**Fig. 3:**
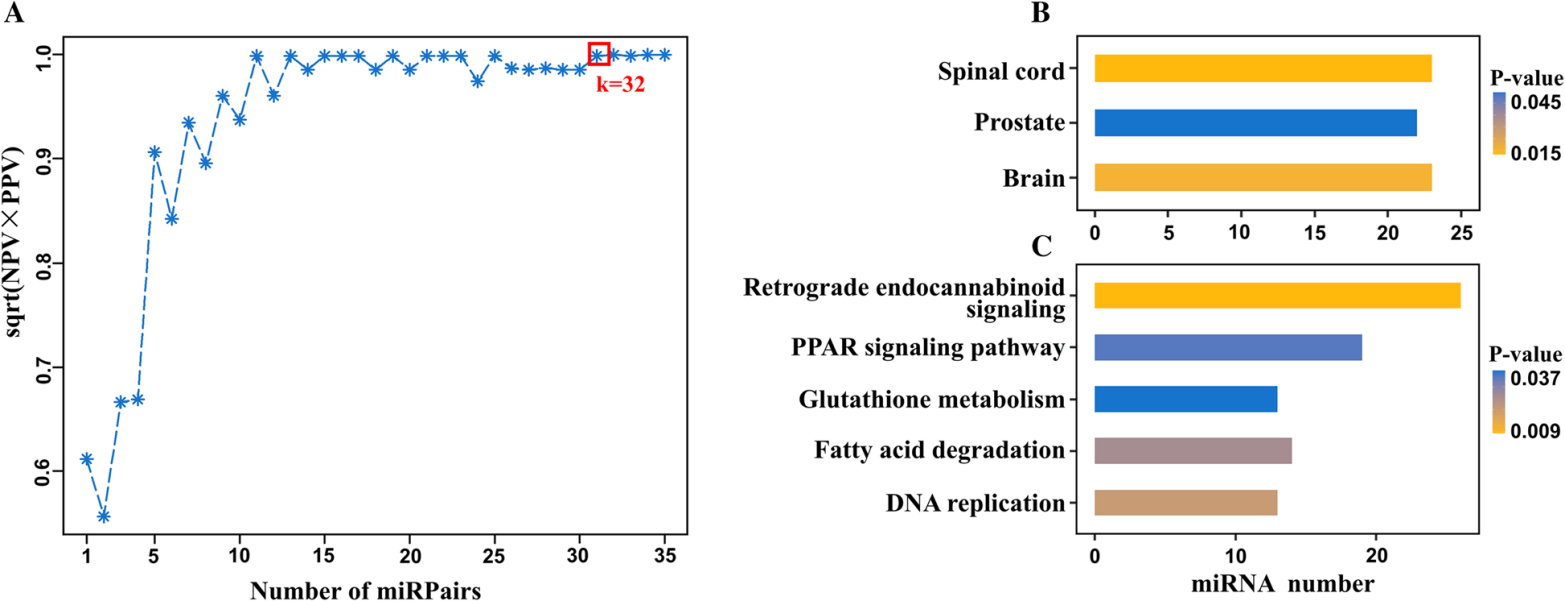
**A** The geometric mean of PPV and NPV of candidate top miRPairs in the training set; **B** The 32-miRPairs associated tissues in Human miRNA tissue atlas database; **C** The 32-miRPairs associated KEGG pathways

To further identify the glioma-specific biomarker, we constructed a model with glioma as the case group and the other cancers as the control (non-glioma) group. The training set GSE113486 contains 13 types of cancers, including 370 cases of bladder cancer and about 40 cases of other cancers. To maintain a balanced sample size, we randomly selected 40 cases of bladder cancer. A total of 1,208,616 stable miRPairs were identified in the non-glioma group when controlling *P*(*E*_*miRNAa*_ *> E*_*miRNAb*_) *≥* 80%. Among them, 753,316 had significantly reversed REOs in the glioma group (FDR < 5%, Fisher’s exact test). Controlling Δ*P* ≥ 0.7 (here Δ*P*=*P*_*non-glioma*_(*E*_*miRNAa*_ *> E*_*miRNAb*_)*-P*_*glioma*_(*E*_*miRNAa*_ *> E*_*miRNAb*_), see Methods), we obtained 1105 candidate miRPairs, and then we searched for the maximum combination of covered samples for each candidate miRPairs. The activity scores were calculated and sorted from largest to smallest. The result showed that the top 32 candidate miRPairs could first classify the training set with 100% accuracy (Fig. 3A). Therefore, they can be used as the serum glioma-specific biomarker, referred to as 32-miRPairs, involving 36 miRNAs (Table S1). A sample was predicted as non-glioma cancer if more than 16 miRPairs exhibited a pattern of *E*_*miRNAa*_ *> E*_*miRNAb*_, and vice versa for glioma samples.

Then, we evaluated the classification efficacy of 32-miRPairs using five independent datasets, namely GSE112264 (*n* = 1252), GSE113740 (*n* = 306), GSE106817 (*n* = 1123), GSE139031 (*n* = 164), and GSE122497 (*n* = 542). The results showed that the prediction accuracy of 32-miRPairs was 98.74, 100, 98.31, 95.73, and 99.45% for the five independent datasets (Table 2), respectively, indicating that 32-miRPairs had reasonable glioma specificity.

### Glioma-specific 32-miRPairs model significantly enriched in brain functional abnormalities

In comparison with glioma samples, we then separately detected the differential miRNAs for the 12 cancer types in training set GSE113486. Results showed that among the 36 miRNAs in 32-miRPairs, an average of 31.25 ± 0.96 was differentially expressed in each cancer type, and 21 were differentially expressed in all cancer types (Fig. 2B).

We conducted the functional enrichment analysis of the 36 miRNAs in 32-miRPairs using the miEAA online miRNA functional enrichment tool, based on the Human miRNA tissue atlas database and the KEGG database. Figure 3B showed that, in the Human miRNA tissue atlas database, these miRNAs could be significantly enriched in the spinal cord (*p* = 0.013) and brain (*p* = 0.015). In the KEGG database, the target mRNAs of these miRNAs were significantly enriched in DNA replication (*p* = 0.021), Fatty acid degradation (*p* = 0.026), Glutathione metabolism (*p* = 0.037), Retrograde endocannabinoid signaling (*p* = 0.009) and PPAR signaling pathway (*p* = 0.035), as shown in Fig. 3C. All of them were previously reported to be associated with glioma [25–28]. The results suggest that the 32-miRPairs may regulate brain-specific miRNA expression.

### Discrimination of glioma from other brain diseases

Considering the enrichment of brain-related functions of the 32-miRPairs, we also collected serum miRNA data to evaluate whether the models can be applied to distinguish glioma from other brain diseases. Two non-neoplastic brain diseases (stroke and Alzheimer’s disease), one benign brain tumor (meningioma), and one malignant brain tumor (PCNSL) were collected. As shown in Table 4, for the stroke dataset, none of the 165 samples were predicted to be glioma by the 5-miRPairs. A similar result was observed for Alzheimer’s disease serum data. Notably, all the healthy controls in these two datasets were also classified correctly, demonstrating the potential of the 5-miRPairs to assist in population screening. All samples for the two neoplastic diseases were predicted as glioma by the 5-miRPairs. When applying the 32-miRPairs, 82.15% of the meningioma and 92.31% of the PCNSL were classified as glioma. This result indicated that the 32-miRPairs might also distinguish neoplastic brain diseases from other cancers and possess brain-specific expression.

**Table 4:** The performance of the predictive models in predicting other brain diseases

GEO accession	Phenotype	Sample No.	5-miRPairs No. (Percentage)^b^	32-miRPairs No. (Percentage)
GSE117064	Healthy	1612(1543)^a^	0(0%)	–
	Stroke	173(165)	0(0%)	–
GSE120584	Healthy	288(277)	0(0%)	–
	Alzheimer’s disease	1021(973)	0(0%)	–
GSE139031	Meningioma	17(16)	16(100%)	13(82.15%)
	PCNSL	42(39)	39(100%)	36(92.31%)

^a^The number in the bracket indicates the remaining sample size after removing outlier samples

^b^The number outside the bracket and the percentages inside the bracket indicate the number and percentage of samples predicted to be positive, respectively

## Discussion

Glioma is highly infiltrative, difficult to remove surgically, and has a poor prognosis [29]. Because of the complex mechanism of glioma development, it is often diagnosed at an advanced stage, and confirming the diagnosis of glioma before the presentation of clinical symptoms remains a significant challenge. Traditional diagnostic techniques mainly include pathologic tissue biopsy and imaging, but both have limitations [3]. Therefore, there is an urgent need for clinically translatable biomarkers that may aid early detection and population screening before clinical symptoms appear. Blood-derived miRNAs serve as promising biomarkers for diagnosing glioma and stratification of glioma subtypes but still have some limitations [30, 31]. We developed a method for detecting robust predictive biomarkers based on the within-sample REOs of serum miRNAs. We identified two panels using 12,447 samples. The first comprised five serum miRNA pairs that are highly accurate in discriminating between glioma and non-cancer control samples. The second consisted of 32 serum miRNA pairs that distinguish glioma from other cancer samples. In addition, we independently validated the two biomarkers in multiple serum cohorts, underscoring their future clinical translational potential for non-invasive detection and population screening of specific glioma.

The REO-based biomarkers were developed by pairing two miRNAs. Compared to single miRNAs, they can resist fluctuations in expression levels [32]. As qualitative biomarkers, they could overcome the drawback of quantitative expression level-based biomarkers. In contrast to the quantitative biomarker, the REO-based biomarkers are insensible to batch effects, data normalization methods, partial RNA degradation, RNA amplification bias, and the proportion of different cancer epithelial cells [13], and thus can be directly applied to individualized clinical diagnosis. The 5-miRPair prediction models would help avoid unnecessary biopsies and could be used in routine screening.

We included the non-cancer samples as controls in the study during the discovery phase to identify biomarkers suitable for early diagnosis. Unlike most studies that compare GBM patients with healthy serum populations, the identified 5-miRPairs are more suitable for clinical application scenarios of cancer detection, i.e., distinguishing whether one has cancer or not, as the early diagnosis population is relatively rarely wholly healthy. By applying to the serum data of stroke and Alzheimer’s diseases, the 5-miRPairs model achieved 100% diagnostic accuracy in these non-neoplastic brain disease samples. For healthy control samples, it still performed well, with none of them classified as glioma. Therefore, our setting of the control population would be suitable for clinical application scenarios of early detection.

One of the data sets we used was derived from Ohno et al. [9]. This study investigated whether miRNAs in serum could detect glioma and distinguish between glioblastoma, primary central nervous system lymphoma, and metastatic brain tumors. In contrast to their study, which focused only on brain tumors, we investigated the potential of serum miRNAs in discriminating glioma from all other cancers. To our knowledge, our study is the first to use large samples for glioma-specific biomarker identification and validation based on within-sample REOs of serum miRNAs. The identified 32-miRPairs achieved high classification accuracy for glioma and other cancers, demonstrating its potential as a glioma-specific biomarker. We have independently validated the identified biomarkers in an extensive sample of serum data to provide evidence for their robustness and utility in a diverse patient population. As these two biomarkers performed well in the training set and numerous validation sets, they could have potential translational utility.

One of the study’s limitations is the lack of stage or grade information in serum glioma samples; thus, we could not directly evaluate the performance of our models in early glioma detection. Another limitation is the lack of data from different platforms. The data used in this study were all from the 3D-Gene Human miRNA V21_1.0.0 platform. However, this should not hinder the potential clinical translation of the developed biomarkers. As discussed by Liu et al., different platforms only affect the number of marker gene pairs and do not affect the diagnostic efficacy of the biomarkers [32]. Our results illustrated that the identified glioma-specific biomarkers could achieve acceptable predictive accuracy even with only a few gene pairs. As shown in Fig. 3A, the geometric mean of NPV and PPV for only five miRPairs was still above 90%.

The number of candidate miRPairs selected for the construction of biomarkers was based on the goals pursued in clinical practice: ease of use and better diagnostic performance. Theoretically, more candidate miRPairs are better. However, as the degrees of reversal of miRPairs (measured by △P) differed, more candidate miRPairs might not necessarily improve the predictive power. For example, the accuracy decreased when using 121 candidate miRPairs for constructing a model discriminating glioma and non-cancer controls. For the glioma-specific model, the accuracy decreased when using 144 candidate miRPairs. Therefore, we chose five candidate miRPairs for the first model, considering the clinical ease of use, as five pairs have already achieved 100% classification accuracy. For the second model, we chose 32 candidate miRPairs because they first reached the maximum of the geometric mean of negative and positive predictive values.

**Fig. 4:**
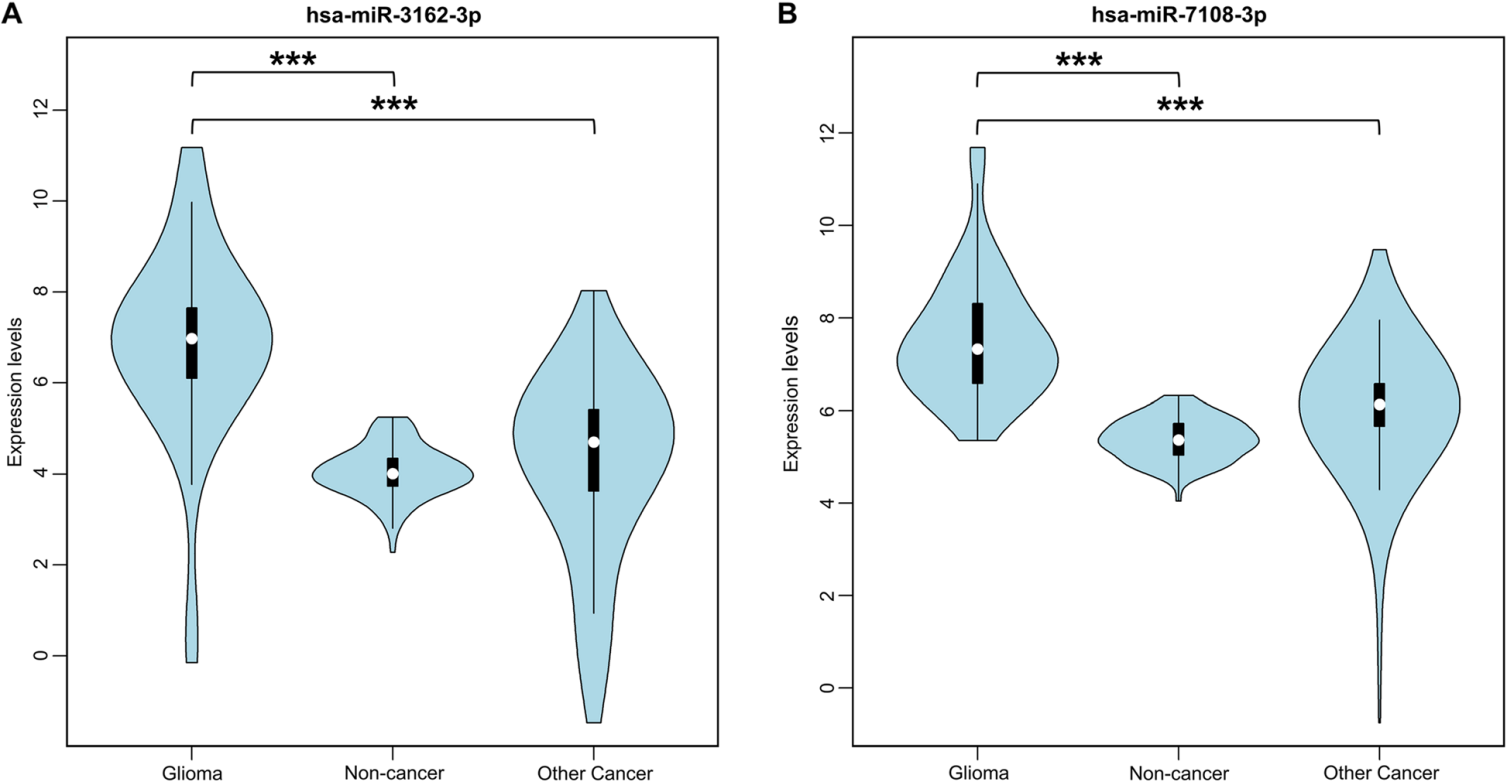
Comparison of expression levels of miRNAs shared by 5-miRPairs and 32-miRPairs. The legend is as following: ***p < 0.001 (Student’s *t*-test)

The 5-miRPairs and 32-miRPairs overlapped two constituted miRNAs which may be attributed to the different settings when constructing the biomarkers. The control samples for constructing 5-miRPairs were non-cancer controls, while the control samples for 32-miRPairs were non-glioma cancer samples. Due to the different settings, the two biomarkers captured potentially different expression features. The 5-miRPairs tended to capture common features of different cancers. Our results have shown that miRNAs in 5-miRPairs were differentially expressed in more than six of the 13 cancers. The 32-miRPairs was inclined to contain differences between gliomas and other cancers, and such differences are more likely to be brain-specific. Thus, these two biomarkers have fewer overlapping miRNAs, with only two, *has-miR-3162-3p* and *has-miR-7108-3p*. Further analysis of the expression of the two miRNAs revealed that, in the training set, they were expressed at the highest levels in glioma samples, lower in the other 12 cancer types, and lowest in non-cancer controls (Fig. 4). Considering the control settings of these two biomarkers, glioma relative to non-cancer controls and glioma relative to other cancer types, such miRNAs could commonly emerge for both application scenarios.

Although we could not find the correlation with glioma from literature for all 36 miRNAs involved in the 32-miRPairs, many were previously reported to be associated with glioma progression. For example, the over-expression of *hsa-miR-134-5p* and *hsa-miR-149* inhibited cell proliferation and migration of glioma cells [33, 34]. The inhibition of *hsa-miR-885-3p* promoted the proliferation and migration of glioblastoma by antagonizing the effects of *HOXB-AS1* knockdown [35]. In glioblastoma, *hsa-miR-495-3p* promotes tumor progression through the spongy action of *LGMN* pseudogene [36]. Of the 36 miRNAs we identified, *hsa-miR-328-3p* [37], *hsa-miR-320b* [38], *hsa-miR-4449* [39], *hsa-miR-346* [40], *hsa-miR-4763-3p* [9], *hsa-miR-133a-3p* [41], *hsa-miR-637* [42] were reported previously as diagnostic or prognostic biomarkers in glioma. These 36 miRNAs were also associated with glioma via multiple pathways such as Retrograde endocannabinoid signaling, DNA replication, Glutathione metabolism, Fatty acid degradation, and PPAR signaling pathways. A striking result was that the 36 miRNAs were directly enriched in the spinal cord and brain tissue-specific expressed miRNAs. As glioma is produced by the brain and spinal cord glial cells [43], 32-miRPairs may regulate brain-specific gene expression, supported by the results that the 32-miRPairs classified most meningioma and PCNSL samples to be glioma. In conclusion, these studies further prove the significance and clinical diagnostic value of the glioma-specific 32-miRPairs.

This study combined all glioma cases into one group. Given the complexity of glioma disease with different types and grades, another more promising utility of non-invasive serum biomarkers lies in determining between high- and low-grade glioma. For example, to distinguish IDH mutant glioma (typically present as low-grade) and IDH wild-type glioblastoma (typically grade four tumors). This issue deserves further attention and will be our study topic when more serum miRNA data are available for different grades of glioma.

## Conclusion

In conclusion, the within-sample relative expression orderings are more suitable and robust than quantitative levels to serve as serum biomarkers for glioma. Upon 12,447 microRNA-profiled serum samples, we identified five and 32 serum microRNA pairs for diagnosis screening and cancer-specific glioma detection with high diagnostic performance in retrospective cohorts. These biomarkers will be prospectively validated to demonstrate their clinical applicability further.

## Supplementary Information

**Additional file 1: Table S1.** Glioma-specific 32-miRPairs.

## Data Availability

The datasets analyzed during the current study are available in GEO (http://www.ncbi.nlm.nih.gov/geo/), accession numbers GSE113486, GSE112264, GSE113740, GSE106817, GSE139031, GSE122497, GSE117064, GSE120584, and GSE 139031.

## Abbreviations

microRNA: miRNA
REO: Relative expression ordering
miRPair: miRNA pair
SEN: Sensitivity
SPE: Specificity
ACC: Accuracy
PPV: Positive predictive value
NPV: Negative predictive value
FDR: False discovery rate
PCNSL: Primary central nervous system lymphoma
