# Supplementary material for: Construct of qualitative diagnostic biomarkers specific for glioma by pairing serum microRNAs
Source: BMC Genomics. 2023 Mar 2;24:96. doi: 10.1186/s12864-023-09203-w (PMC9983174; doi:10.1186/s12864-023-09203-w)
Supplement: Supplementary file 1 — Additional file 1: Table S1. Glioma-specific 32-miRPairs. [file 12864_2023_9203_MOESM1_ESM.docx]

Table S1 Glioma-specific 32-miRPairs

| **miRNAa** | **miRNAb** | **Ratio_1_^†^** | **Ratio_2_^‡^** | **P-value** | **Activity score** |
| --- | --- | --- | --- | --- | --- |
| hsa-miR-134-5p | hsa-miR-4727-5p | 0.882 | 0.132 | 2.34E-19 | 326 |
| hsa-miR-134-5p | hsa-miR-516a-5p | 0.865 | 0.158 | 2.54E-17 | 207 |
| hsa-miR-4727-3p | hsa-miR-6070 | 0.974 | 0.237 | 2.69E-24 | 204 |
| hsa-miR-373-5p | hsa-miR-4665-3p | 0.900 | 0.184 | 1.62E-18 | 109 |
| hsa-miR-371a-5p | hsa-miR-4665-3p | 0.956 | 0.237 | 8.52E-22 | 99 |
| hsa-miR-149-3p | hsa-miR-4763-3p | 0.863 | 0.053 | 1.42E-21 | 83 |
| hsa-miR-557 | hsa-miR-4792 | 0.865 | 0.158 | 2.54E-17 | 76 |
| hsa-miR-4727-3p | hsa-miR-585-5p | 0.913 | 0.158 | 1.40E-20 | 70 |
| hsa-miR-320b | hsa-miR-636 | 0.867 | 0.158 | 1.96E-17 | 62 |
| hsa-miR-320b | hsa-miR-516a-5p | 0.980 | 0.237 | 1.17E-25 | 61 |
| hsa-miR-4727-3p | hsa-miR-516a-5p | 0.959 | 0.237 | 4.60E-22 | 60 |
| hsa-miR-320b | hsa-miR-3622b-3p | 0.969 | 0.263 | 1.60E-22 | 53 |
| hsa-miR-557 | hsa-miR-7108-3p | 0.843 | 0.105 | 4.60E-18 | 44 |
| hsa-miR-5572 | hsa-miR-941 | 0.976 | 0.263 | 1.45E-23 | 37 |
| hsa-miR-134-5p | hsa-miR-6869-3p | 0.839 | 0.132 | 7.01E-17 | 32 |
| hsa-miR-4449 | hsa-miR-4792 | 0.911 | 0.184 | 2.46E-19 | 32 |
| hsa-miR-4727-3p | hsa-miR-31-3p | 0.906 | 0.158 | 4.86E-20 | 32 |
| hsa-miR-4433a-3p | hsa-miR-4665-3p | 0.946 | 0.132 | 5.45E-25 | 31 |
| hsa-miR-320b | hsa-miR-585-5p | 0.954 | 0.237 | 1.60E-21 | 28 |
| hsa-miR-320b | hsa-miR-133a-3p | 0.952 | 0.211 | 2.61E-22 | 27 |
| hsa-miR-4658 | hsa-miR-516a-5p | 0.867 | 0.158 | 1.96E-17 | 25 |
| hsa-miR-4727-3p | hsa-miR-3162-3p | 0.863 | 0.026 | 6.69E-23 | 25 |
| hsa-miR-557 | hsa-miR-6729-3p | 0.806 | 0.105 | 2.71E-16 | 24 |
| hsa-miR-320b | hsa-miR-31-3p | 0.946 | 0.184 | 1.35E-22 | 20 |
| hsa-miR-134-5p | hsa-miR-6070 | 0.893 | 0.132 | 4.02E-20 | 19 |
| hsa-miR-885-3p | hsa-miR-637 | 0.874 | 0.158 | 8.43E-18 | 19 |
| hsa-miR-328-3p | hsa-miR-516a-5p | 0.902 | 0.132 | 8.35E-21 | 17 |
| hsa-miR-3151-5p | hsa-miR-3162-3p | 0.821 | 0.053 | 3.83E-19 | 17 |
| hsa-miR-4727-3p | hsa-miR-3177-5p | 0.863 | 0.132 | 3.86E-18 | 17 |
| hsa-miR-4487 | hsa-miR-495-3p | 0.915 | 0.184 | 1.09E-19 | 16 |
| hsa-miR-4485-5p | hsa-miR-941 | 0.978 | 0.211 | 3.53E-26 | 16 |
| hsa-miR-346 | hsa-miR-516a-5p | 0.839 | 0.132 | 7.01E-17 | 15 |

**^†^**Ratio1 represents the ratio of E_miRNAa_>E_miRNAb_ in non-glioma.

**^‡^**Ratio2 represents the ratio of E_miRNAa_>E_miRNAb_ in glioma.
